# Stratification From Heterogeneity of the Cell-Death Signal Enables Prognosis Prediction and Immune Microenvironment Characterization in Esophageal Squamous Cell Carcinoma

**DOI:** 10.3389/fcell.2022.855404

**Published:** 2022-04-12

**Authors:** Yiyuan Zhang, Yanxing Chen

**Affiliations:** ^1^ Department of Radiation Oncology, Shandong First Medical University and Shandong Academy of Medical Sciences, Jinan, China; ^2^ Department of Radiation Oncology, Shandong Cancer Hospital and Institute, Shandong First Medical University and Shandong Academy of Medical Sciences, Jinan, China; ^3^ State Key Laboratory of Oncology in South China, Department of Medical Oncology, Sun Yat-sen University Cancer Center, Collaborative Innovation Center for Cancer Medicine, Guangzhou, China

**Keywords:** cell-death signals, esophageal squamous cell carcinoma, heterogeneity, prognosis, immune microenvironment

## Abstract

Esophageal squamous cell carcinoma (ESCC) is the primary subtype of esophageal cancer (EC) characterized by a high incidence rate and extremely poor prognosis worldwide. Previous studies suggested that the specific cell death signal was linked to different immune subtypes in multiple cancers, while a comprehensive investigation on ESCC is to be performed yet. In the current study, we dissected different cell death signals in ESCC tumors and then integrated that functional information to stratify ESCC patients into different immunogenic cell death (ICD) subtypes. By systematically analyzing the transcriptomes of 857 patients and proteomic profile of 124 patients, we found that the signals of necroptosis, pyroptosis, and ferroptosis are positively associated with activated immunity in ESCC. We identified two ICD pattern terms, namely, ICD-high and ICD-low subtypes that positively correlated to both progression-free survival and overall survival. In addition, cell fraction deconvolution analysis revealed that more infiltrated leukocytes were enriched in ICD-high types, especially antigen-presenting cells, such as dendritic cells and macrophages. With the XGBoost algorithm, we further developed a 14-gene signature which can simplify the subtyping for allocating new samples, by which we validated the prognosis value of the signature and proved that the ICD score scheme could serve as a promising biomarker for stratifying patients with immunotherapy in several immune checkpoint blockade treatment cohorts. Collectively, we successfully constructed the ICD scheme, which enables predicting of the prognosis or immunotherapy efficacy in ESCC patients and uncovered the critical interplay between cell death signals and immune status in ESCC.

## Introduction

Esophageal cancer (EC) is the 10th common cancer worldwide, while its mortality ranks sixth among all types of cancers (Sung et al., 2021). Esophageal squamous cell carcinoma (ESCC) accounts for nearly 90% of all the incident esophageal cancers each year, and regions of high incidence of ESCC include Eastern to Central Asia (Abnet et al., 2018). Even though great effort has been devoted to investigating its molecular characteristic (Cancer Genome Atlas Research, 2017) effectively, a therapeutic strategy against metastasis ESCCs was still limited until the development of immunotherapy. Recently, immunotherapy combined with chemotherapy has been proven effective in the first-line treatment of ESCC (Luo et al., 2021; Sun et al., 2021), which indicates that the therapeutic strategy of metastasis ESCC is stepping into a new era. Interestingly, different chemotherapy methods were chosen in these two clinical trials, among which Luo et al. (Luo et al., 2021) used Taxol plus platinum-based chemotherapy, while Sun et al. (Sun et al., 2021) used fluoropyrimidine plus platinum-based chemotherapy. As the efficacy of the former used in all patients is comparable to the latter used in high-PD-L1 patients, how to choose optimal chemotherapy for combinations based on the mechanism remains to be clarified.

As up to 60% of solid cancer patients do not respond to single-agent ICI therapy, the combination strategy breaks new ground for the use of immunotherapy (Gandhi et al., 2018; Paz-Ares et al., 2018; Galsky et al., 2020; Shitara et al., 2020). Despite huge success made in clinical trials, the mechanisms of how chemotherapy enhancing immunotherapy are to be fully understood yet, which would make choosing the optimal combination strategy a huge challenge.

Recently, scientists found that chemotherapy can induce immunogenic cell death (ICD) of tumor cells (Galluzzi et al., 2020), which could significantly enhance the anti-tumor immunity in a tumor microenvironment. However, different types of cell death have different effects on anti-tumor immunity (Legrand et al., 2019). For example, intrinsic apoptosis induced by mitochondrial outer membrane permeabilization allows the host to quickly and efficiently clear away dead cell corpses without triggering an immune response (Arandjelovic and Ravichandran, 2015). Autophagy could suppress antigen processing and presentation of tumor, and inhibition of autophagy may enhance immunotherapy (Deng et al., 2021; Thorburn and Towers, 2021). On the contrary, necroptosis provoked by TNF signaling of IFN stimulation is strongly correlated with activated immunity (Newton et al., 2016), which ensures that a powerful alert message is sent to the immune system. Similarly, pyroptosis is also an immunogenic cell death since its hyperinflammatory nature through IL-1 secretion and DAMP release could trigger inflammatory responses in the tumor microenvironment and increase leukocyte infiltration (Legrand et al., 2019). A recent research study also showed that tumor ferroptosis could be promoted by CD8^+^ T cell (Wang et al., 2019a), which proved that ferroptosis is another type of cell death that is positively associated with activated anti-tumor immunity. As the types and levels of cell death do have huge difference across different tumors, subtyping tumors with a cell death signal could help us characterize cancer patients concerning cell death and subsequently uncover the connection across cell death, immune microenvironment, and clinical features. Moreover, the subtyping would further help us choose specific agents that could induce specific cell death with high immunogenicity, which is an important step for making the most of an immunotherapy combination strategy.

Specific cell death signatures and cell death subtyping such as pyroptosis-related signature (Ye et al., 2021), pyroptosis-related subtyping (Song et al., 2021), and immunogenomic gene signature of cell-death-associated genes (Ahluwalia et al., 2021) have been proven that they could stratify cancer patients into different subgroups that are strongly associated with clinical features and immune microenvironment. However, none of this research combined different cell death signals together to characterize the tumors and systematically subtype the tumors concerning all immunogenic cell death in different omics levels. Here, with the transcriptome data of 857 patients and proteomic data of 124 patients, we found that signals of necroptosis, pyroptosis, and ferroptosis were positively associated with activated immunity in ESCC and subsequently defined ICD-high and ICD-low subtypes of the ESCCs with these three signals in both transcriptomic and proteomic levels. The subtyping could not only help us systematically understand the connection between cell death and immunity in ESCC but also help predicting the prognosis of ESCC patients and immunotherapy efficacy.

## Methods

### ESCC Data Source and Preprocessing

A total of seven ESCC microarray cohorts (GSE53625 (Li et al., 2014), GSE23400 (Su et al., 2011), GSE38129 (Hu et al., 2015), GSE47404 (Sawada et al., 2015), GSE69925 (Tanaka et al., 2015), GSE121931 (Li et al., 2020), and GSE44021 (Yang et al., 2020)) were collected from the GEO database (https//www.ncbi.nlm.nih.gov/geo/) (Table 1). A total of three GEO datasets (GSE53625, GSE23400, and GSE38129) were enrolled to perform a comparison between tumor and normal samples, with the standard that the number of tumor/normal sample pairs from the identical dataset and the identical platform was no less than 30. In total, 262 paired tumor and normal samples were merged. The tumor transcriptomics cohort consists of 775 tumor samples from the aforementioned GEO datasets, with the standard that the number of tumor samples from the sample dataset and the identical platform was no less than 50. Non-biological technical biases were corrected by “Combat” function of the sva R package (Leek et al., 2012). Mutation, copy number variation, and transcriptome data of ESCC from TCGA database were downloaded from the UCSC Xena datahub (https://xenabrowser.net/datapages/). The proteomic data and corresponding clinical data for 124 ESCC samples were obtained from Liu et al.‘s study(Liu et al., 2021). In total, four published immunotherapy datasets were collected to support the predictive value of ICD-score in immune checkpoint blockade treatment, including three SKCM cohorts and 1 urothelial carcinoma. (Van Allen et al., 2015; Mariathasan et al., 2018; Gide et al., 2019; Liu et al., 2019).

**TABLE 1:** Summary of all datasets in this study.

Data source	Dataset	Number of normal	Number of tumor
GEO	GSE53625	179	179
	GSE23400-GPL96	53	53
	GSE38129	30	30
	GSE69925	0	274
	GSE121931	0	125
	GSE44021-GPL571	73	73
	GSE47404	0	71
TCGA	TCGA-ESCC	0	91
Liu et al.‘s study	Proteomics data	0	124

### Immunogenic Cell-Death Pathway and Consensus Cluster

We summarized five cell death pathways which are apoptosis, autophagy, ferroptosis, necroptosis, and pyroptosis from the GO database (http://geneontology.org/), KEGG database (https://www.genome.jp/kegg/pathway.html), and Reactome database (https://reactome.org/). Gene set variation analysis (GSVA) was used to evaluate the relative activity of these cell death pathways (Hänzelmann et al., 2013). By comparing the correlation between immune-associated pathways and five cell death pathways, we selected the three most related pathways (ferroptosis, necroptosis, and pyroptosis), termed as the immunogenic cell death (ICD) pathway, to perform the clustering analysis. An unsupervised clustering analysis was applied to identify distinct cell death pathway activity patterns based on the GSVA score of the ICD pathway and classify patients for further analysis (Monti et al., 2003). The number of clusters and their stability was determined by the consensus clustering algorithm. The ConsensusCluserPlus package was utilized to perform the aforementioned steps, and 1,000 times repetitions were set to guarantee the robustness of clustering. The PCA algorithm was used to assess heterogeneity between different clusters.

### Estimation of TME Cell Infiltration

We quantify the relative abundance of different immune cells in the ESCC microenvironment by GSVA. The marking gene set of each infiltration immune cell was obtained from Dvir Aran et al.‘s study (Aran et al., 2017). Quantification of tumor microenvironment signals including lymphocyte infiltration, macrophages, TGF-beta, IFN-gamma, and wound healing was performed on transcriptomic data of ESCC samples according to Thorsson et al. (Thorsson et al., 2018a).

### Construction of the ICD-Score

On the basis of the most important genes in the ICD pathway, we constructed the ICD-score. Specifically, we first take an intersection of the ICD gene between transcriptomics data and protein data. Next, we sort the overlapping genes by the Extreme Gradient Boosting (XGBoost) algorithm according to the importance of distinguishing ESCC patients into ICD-high or ICD-low clusters. XGBoost is one of the most widely used tree-based boosting algorithms, in which a set of weak classifiers is combined to form a strong classifier sequentially (Chen and Guestrin, 20162016). In each iteration, misclassification errors of a previous classifier were corrected by the next classifier to perform more accurately. More importantly, it can avoid overfitting by effective ways. Importance is applied to access the contribution of each variable to the classification, which is measured for a single decision tree by the amount that each attribute split point improves the performance measure. The importance of each variable is the average of all decision trees. We selected top 30% genes ranked by importance to construct the ICD-score by calculating their arithmetic mean. We build the protein–protein interaction network of 14 genes with the STRING (https://www.string-db.org/) database and IMEx database (Breuer et al., 2013) in NetworkAnalyst (Zhou et al., 2019).

### Statistical Analysis

The correlation coefficient between two numeric variables’ cell death was calculated using the Spearman coefficient. The Wilcoxon rank-sum test was used to compare the differences of numeric variables between two groups. Univariate Cox regression was applied to evaluate the relationship between different subgroups and prognosis. Multivariate Cox regression was used to identify independent prognostic factors. Multi-factor Cox regression results were visualized by the ‘forestplot’ R package. All statistics *p* values were two-tailed, and *p* < 0.05 was regarded as statistically significance. All data were processed using R 4.1.0.

## Result

### Identification of the Immunogenic Cell Death Pathway in ESCC

**FIGURE 1:**
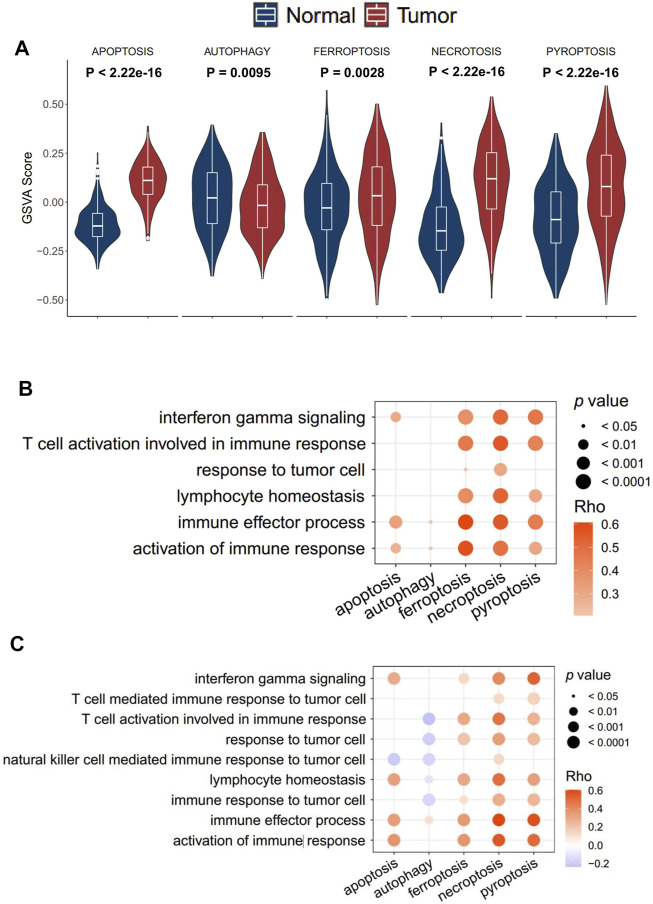
Identification of the immune cell death pathway. **(A)** GSVA score of cell death signaling between tumor and normal tissue in the transcriptomics level; **(B,C)** correlation of five cell death signaling and immune responding-related signaling in the transcriptomics level **(B)** and proteomics level **(C)**.

In total, five cell death pathways and the related genes were collected from a public database for the analysis, including apoptosis (234 genes), autophagy (169 genes), ferroptosis (40 genes), necroptosis (40 genes), and pyroptosis (50 genes) (Supplementary Table S1). The activity of apoptosis, ferroptosis, necroptosis, and pyroptosis in tumor tissue was significantly higher than that in normal tissue, while the difference of apoptosis was moderate. More interestingly, the autophagy pathway showed a reverse trend (Figure 1A). The result revealed that differences exist between normal tissue and tumor tissue concerning the types of cell death. In the transcriptomic level, a significantly positive correlation was observed between ferroptosis, necroptosis, pyroptosis, and immune-associated pathways, such as immune effector process and activation of immune response (Figure 1B). On the contrary, apoptosis and autophagy showed no significantly positive correlation with immune response signals. The result was further validated in the proteomics level (Figure 1C). Accordingly, only ferroptosis, necroptosis, and pyroptosis have a strong correlation with immune response signals, and we consequently defined the three cell death pathways as immune-associated cell death (ICD) pathways.

### The Landscape of Genetic Variation of Cell Death Pathways in ESCC

**FIGURE 2:**
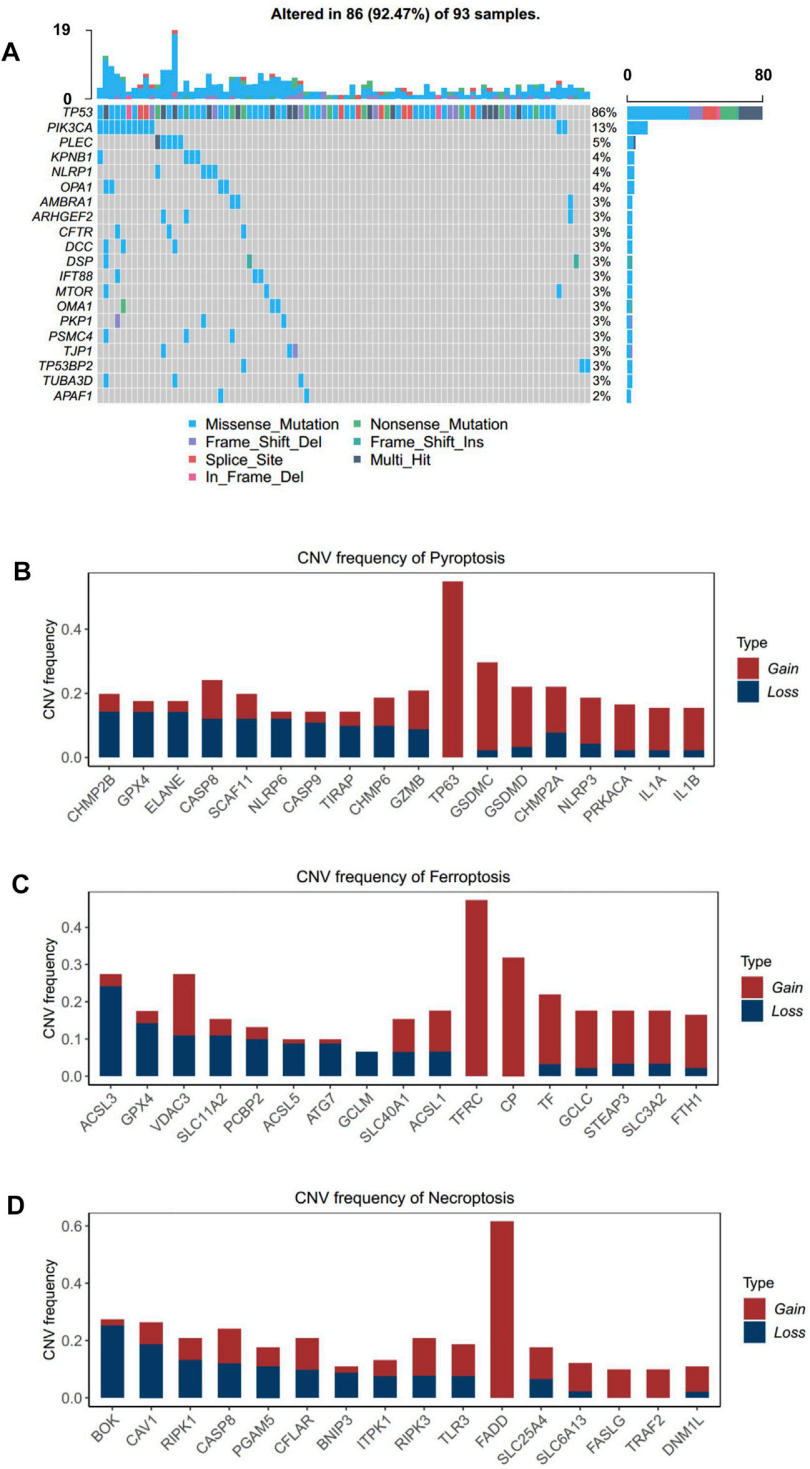
Landscape of genetic alterations of cell death genes in ESCC. **(A)** Mutation frequency of cell death genes in 91 patients with ESCC from the TCGA-ESCC cohort. Upper: the bar plot shows the number of mutant genes in individual patients. Right: the bar plot indicates the proportion of each variant type. **(B–D)**. CNV frequency of pyroptosis, **(B)** ferroptosis, and **(C)** necroptosis **(D)** in the TCGA-ESCC cohort. The height of the column represents the alteration frequency. Variation color indicates the class of CNV (red for amplifications and blue for deletions).

In order to investigate the genomic alteration in cell death pathways, we summarized the incidence of somatic mutation and copy number variation (CNV) concerning the corresponding genes in TCGA-ESCC cohort. Among 93 ESCC patients, genetic mutation of ICD pathways occurred in 86 patients, with a frequency of 92.47%, primarily including missense mutation, nonsense mutation, and splice site. Ranked by mutation frequency, TP53, a well-known tumor suppressor gene (Olivier et al., 2010; Fromentel and Soussi, 1992), is the most prevalently mutated genes among all the ICD genes. PIK3CA and PLEC were the following two genes, with frequencies of 13 and 5%, respectively (Figure 2A). As ESCC is a tumor with a high copy number variation (CNV) burden (Cui et al., 2020), CNV also occurs frequently on ICD genes. Specifically, TP63 in pyroptosis, TFRC in ferroptosis, and FADD in necroptosis presented the highest frequency of amplification, while CHMP2B, GPX4, and ELANE in pyroptosis, ACSL3 in ferropotosis, and BOK in necroptosis had the highest frequency of deletion (Figures 2B–D). In addition, CNV of different genes had different effects on the activity of corresponding cell death pathways. CHMP2B, TF, and ITPK1 were three genes whose copy numbers were most positively correlated with the respective pathways, while NLRP3, GCLM, and FASLG were three genes whose copy numbers were most positively correlated with the respective pathways (Supplementary Figure S1A). The result showed that genomic alteration of ICD genes was common in ESCC, which implied that the irregulation of ICD pathways may contribute to the tumorigenesis and development ESCC.

### Subtyping ESCCs With the Signal of ICD Pathways

**FIGURE 3:**
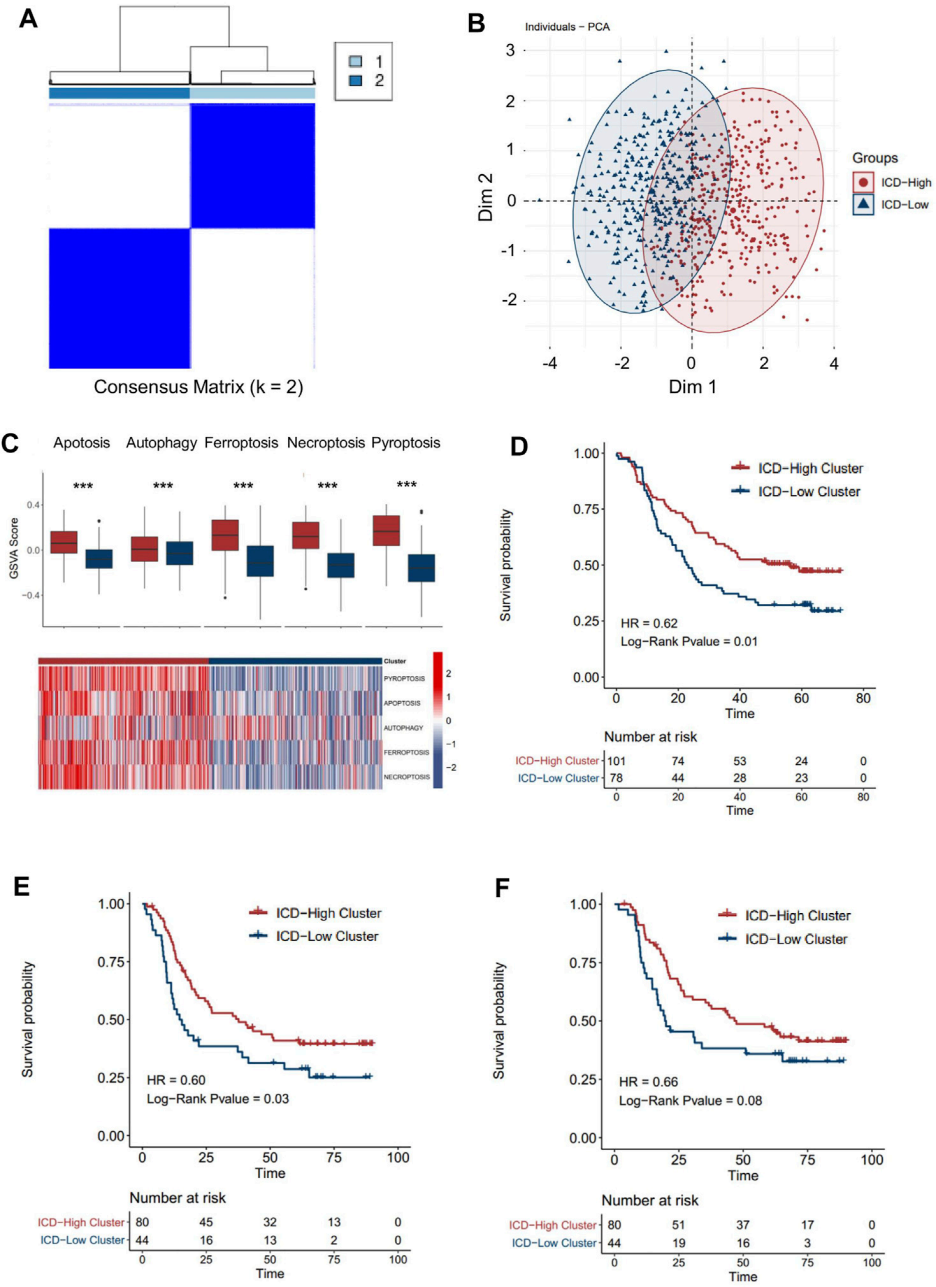
Patterns of the immune cell death pathway and prognosis characteristics of each pattern. **(A)** Unsupervised clustering of three immune cell death pathways’ GSVA scores in the transcriptomics level. **(B)** PCA analysis for two ICD clusters in the transcriptomics level. **(C)** Cell death pathway patterns of two ICD clusters in the transcriptomics level. Top: the bar plot shows the cell death pathway GSVA score of two ICD clusters. Bottom: the heatmap reveals the GSVA score pattern of two ICD clusters **(D–F)**. Kaplan–Meier analysis of overall survival (OS) or progression-free survival (PFS) comparing the ESCC patients with two ICD clusters. **(D)** Kaplan–Meier analysis of OS in the transcriptomics level. **(E)** Kaplan–Meier analysis of PFS in the transcriptomics level. **(F)** Kaplan–Meier analysis of OS in the proteomics level (red for the ICD-high cluster; blue for the ICD-low cluster).

A consensus clustering analysis based on ICD pathway GSVA scores was performed on the integrated dataset after batch effect removal (Supplementary Figures S2A,B). After selecting the optimal k value (Figure 3A, Supplementary Figure S3A), two distinct ICD patterns were eventually identified in the transcription level (Figure 3A). PCA analysis also revealed the heterogeneity of the two groups concerning cell death signals (Figure 3B). An identical phenomenon was also observed in the proteomics dataset (Supplementary Figures S4A,B). By comparing the relative level of the cell death signal, we found that ferroptosis, necroptosis, and pyroptosis signals were extremely variant between the two groups (Figure 3C, Supplementary Figure 4C). Thus, we defined the group with higher ICD pathway signals as an ICD-high cluster, and the other group as an ICD-low cluster.

By integrating the clinical data, we found that ESCC patients in the ICD-high cluster had a better overall survival than those in the ICD-low cluster (Figure 3D, HR = 0.62, *p*-value = 0.01) in the transcriptomic cohort. Similarly, longer progression-free survival (Figure 3E, HR = 0.62, *p*-value = 0.03) and overall survival (Figure 3F, HR = 0.62, *p*-value = 0.08) were also detected in ICD-high patients than in ICD-low patients in the proteomic level, which further validated the prognostic relevance of the ICD subtyping.

### The Immune Characteristic of Two ICD-Associated Patterns

**FIGURE 4:**
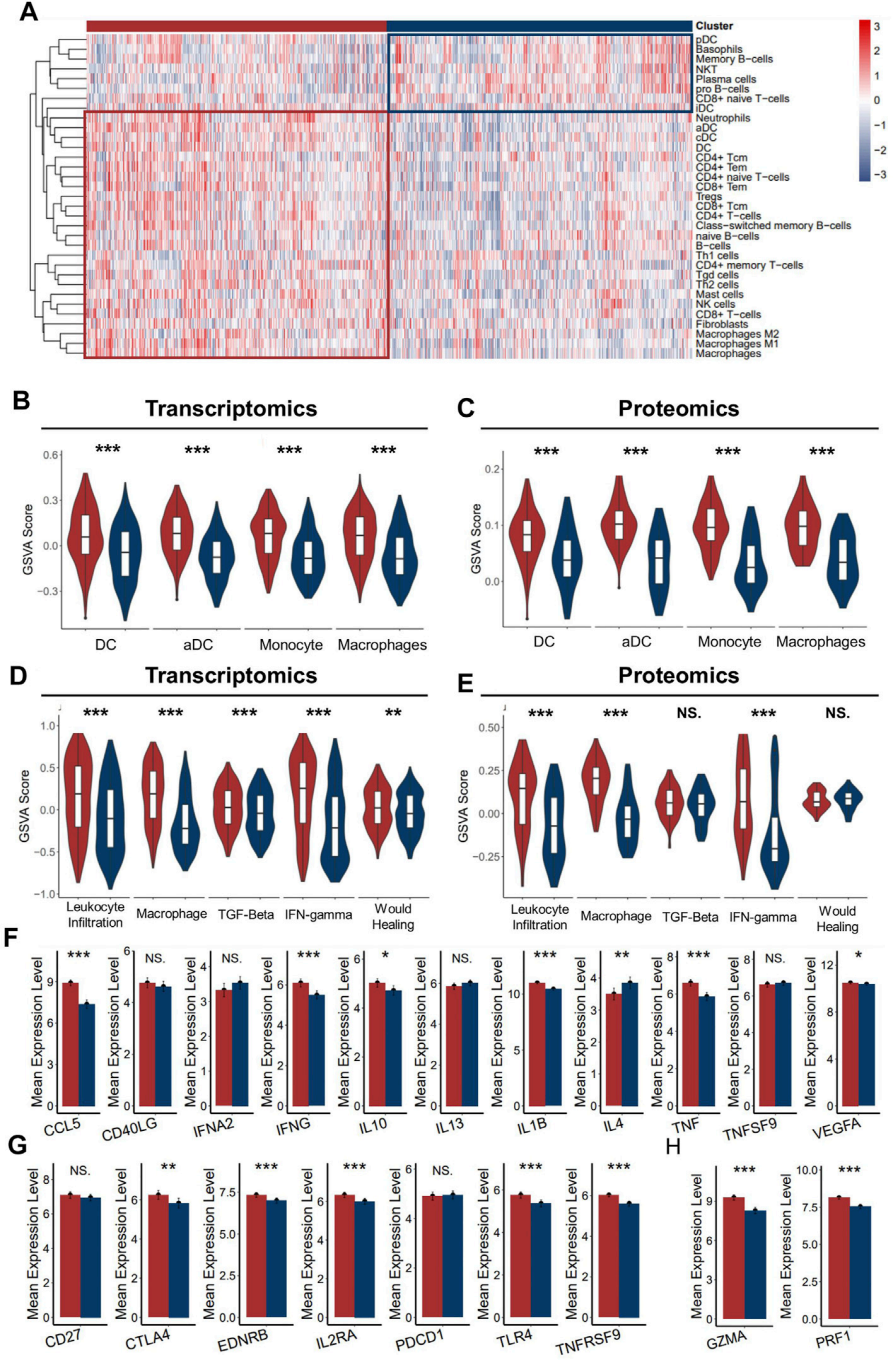
Immune infiltration characteristics of two ICD clusters. **(A)** Heatmap presents the filtration of immune cells in two ICD clusters **(B,C)**. The enrichment of major antigen-presenting cells of two clusters in the transcriptomics level **(B)** and proteomics level **(C–E)**. GSVA scores of five immune signatures of two ICD clusters in the transcriptomics level **(D)** and proteomics level **(E,F)**. The mean expression of immunoregulator ligands of two ICD clusters in the transcriptomics level. **(G)** Mean expression of immunoregulator receptors of two ICD clusters in the transcriptomics level. **(H)** Mean expression of cytotoxic activity signature of two ICD-score clusters in the transcriptomics level. (Mann–Whitney *U* test: NS: no statistical difference, *: *p* < 0.05, **: *p* < 0.01, ***: *p* < 0.0001) (red for the ICD-score high cluster; blue for the ICD-score low cluster).

As better prognosis was detected in ICD-high patients, we speculated that the strong correlation with anti-tumor immunity might be one of the explanations for the clinical relevance. To further clarify the tumor immune microenvironment between the two groups in detail, we assessed the level of infiltrated immune cell, immune subtyping signals, and immunomodulator genes in these two groups. As expected, the abundance of most infiltrated immune cells was significantly higher in the ICD-high cluster than in the ICD-low cluster (Figure 4A), which was validated with the proteomics dataset (Supplementary Figure S5A). Specifically, we noticed that myeloid cells, such as dendritic cells, neutrophils, monocytes, and macrophages were the most upregulated cell types in the ICD-high cluster (Figures 4B,C). It is well known that dendritic cells and macrophages are the main antigen-presenting cells among the immune reaction. As ferroptosis, necroptosis, and pyroptosis were reportedly associated with immune activation in the tumor microenvironment, we assumed that the higher level of neoantigen presentation activity might have occurred in the ICD-high cluster, probably due to more release of the immunogenic tumor neoantigen. Vesteinn Thorsson et al. (Thorsson et al., 2018b) categorized cancer immunity into six subtypes by five signatures (lymphocyte infiltration, macrophages, TGF-beta, IFN-gamma, and wound healing). As expected, signals directly concerning the immune reaction were significantly higher in the ICD-high cluster, especially macrophage signals (Figures 4D,E). In addition, we also assessed the expression level of immunomodulator genes between the two groups. Both immunomodulatory ligands and receptors tend to have a higher expression level in the ICD-high group than in the ICD-low group (Figures 4F,G). In addition, the expressions of markers of cytotoxic activity were also prevailing in the ICD-high cluster (Figure 4H), suggesting a more activated anti-tumor immunity in ICD-high patients. Conclusively, these results implied that the ICD-high cluster has a relatively hotter tumor immune environment.

### Construction of ICD-Score and Investigation of Its Biological Relevance

**FIGURE 5:**
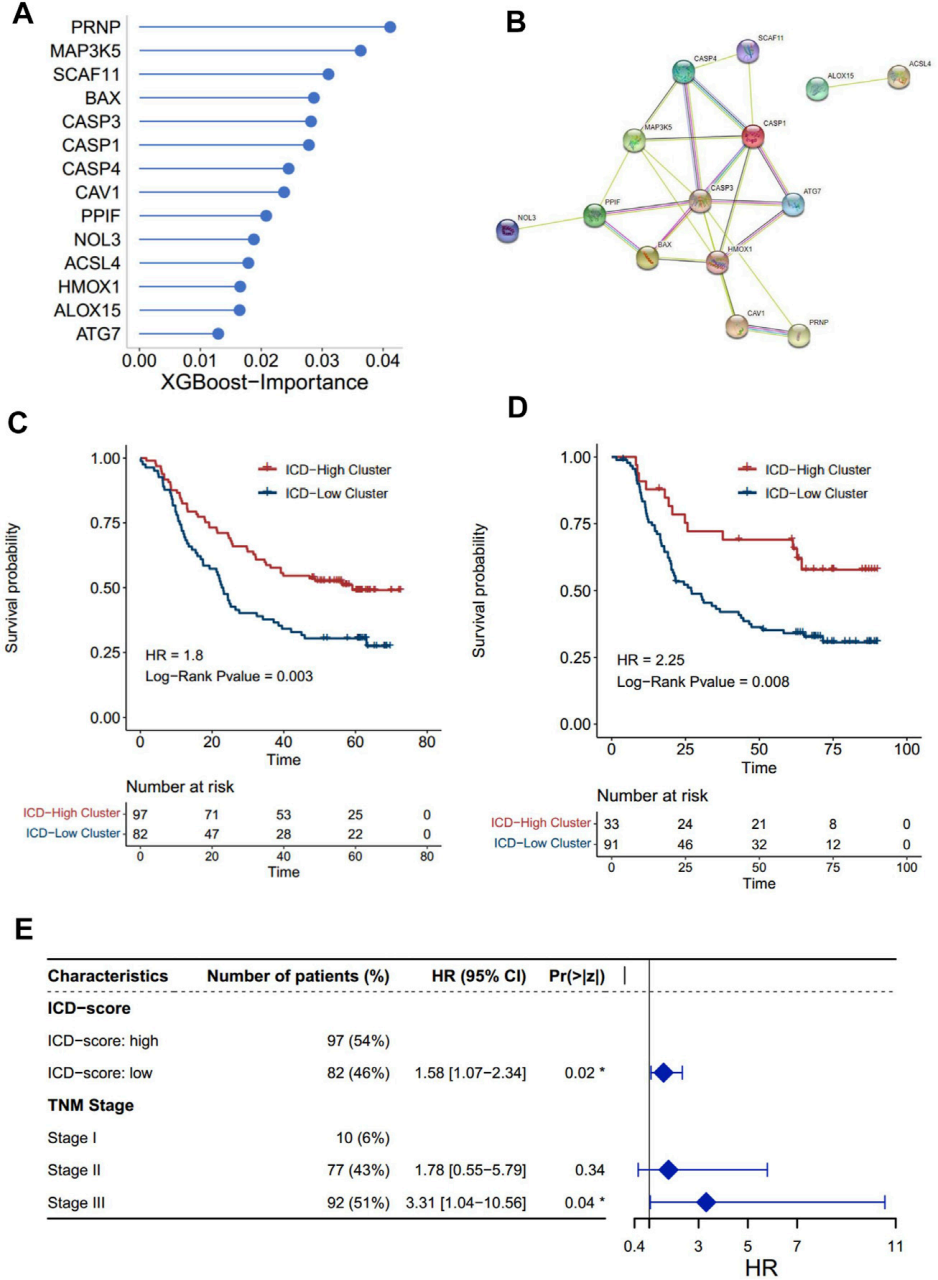
Construction of the ICD score and prognosis characteristics. The importance of 14 genes calculated by the XGBoost algorithm. **(A)** Higher importance value of a gene indicated higher importance. **(B)** Protein–protein interaction network of 14 proteins built by the STRING database. The nodes represent the proteins; the line represents the relationship of proteins. **(C,D)** Kaplan–Meier analysis of overall survival (OS) comparing the ESCC patients with the two ICD score clusters. **(C)** Kaplan–Meier analysis of OS in the transcriptomics level. **(D)** Kaplan–Meier analysis of OS in the proteomics levels (red for the ICD-high cluster; blue for the ICD-low cluster). **(E)** Forest plot: Cox multivariate mode of the ICD-score and the TNM stage in the transcriptomics level.

Our findings have already shown the potential role of the ICD pathway in prognosis and immune infiltration modulation. To further push forward the use of the subtyping of the ICD signal, denoting the subtyping with a brief gene signature would be more helpful. Thus, on the basis of the ICD genes, we constructed a set of scoring systems to quantify the ICD pathway pattern of individual ESCC patients, termed as the ICD-score. In order to select optimal features to construct a more concise model, we used the XGBoost algorithm, which has been proved to be better than other machine learning algorithms on classification and feature selection in several research studies (Huang et al., 2020; Le et al., 2020; Chen et al., 2021). Accordingly, we obtained 14 genes to profile the ICD-score (Figure 5A), among which PRNP was the most important gene, followed by MAP3K5 and SCAF11. A protein–protein intersection network built by different databases revealed the tight connection between the 14 proteins (Figure 5B, Supplementary Figure S6A). Similar to subtyping, the ICD-score was also positively correlated with infiltration of myeloid cells in charge of neoantigen presentation, such as dendritic cells, neutrophils, monocytes, and macrophages (Supplementary Figure S6B). As expected, the ICD-score low cluster had a worse overall survival rate (Figures 5C,D). Adjusting the influence of the TNM stage by multi-variable Cox regression, we found that the ICD-score can act as an independent factor to predict the prognosis of ESCC patients (transcriptomics: log-rank *p* = 0.02, HR = 1.58 [1.07-2.34]; proteomics: log-rank *p* = 0.02, HR = 2.08[1.12-3.85]) (Figure 5E, Supplementary Figure S6C). Our analysis suggested that the ICD-score model can represent ICD patterns and act as an independent factor in predicting the prognosis of ESCC patients.

### Correlation Between Oncogenic Alteration and ICD-Score

**FIGURE 6:**
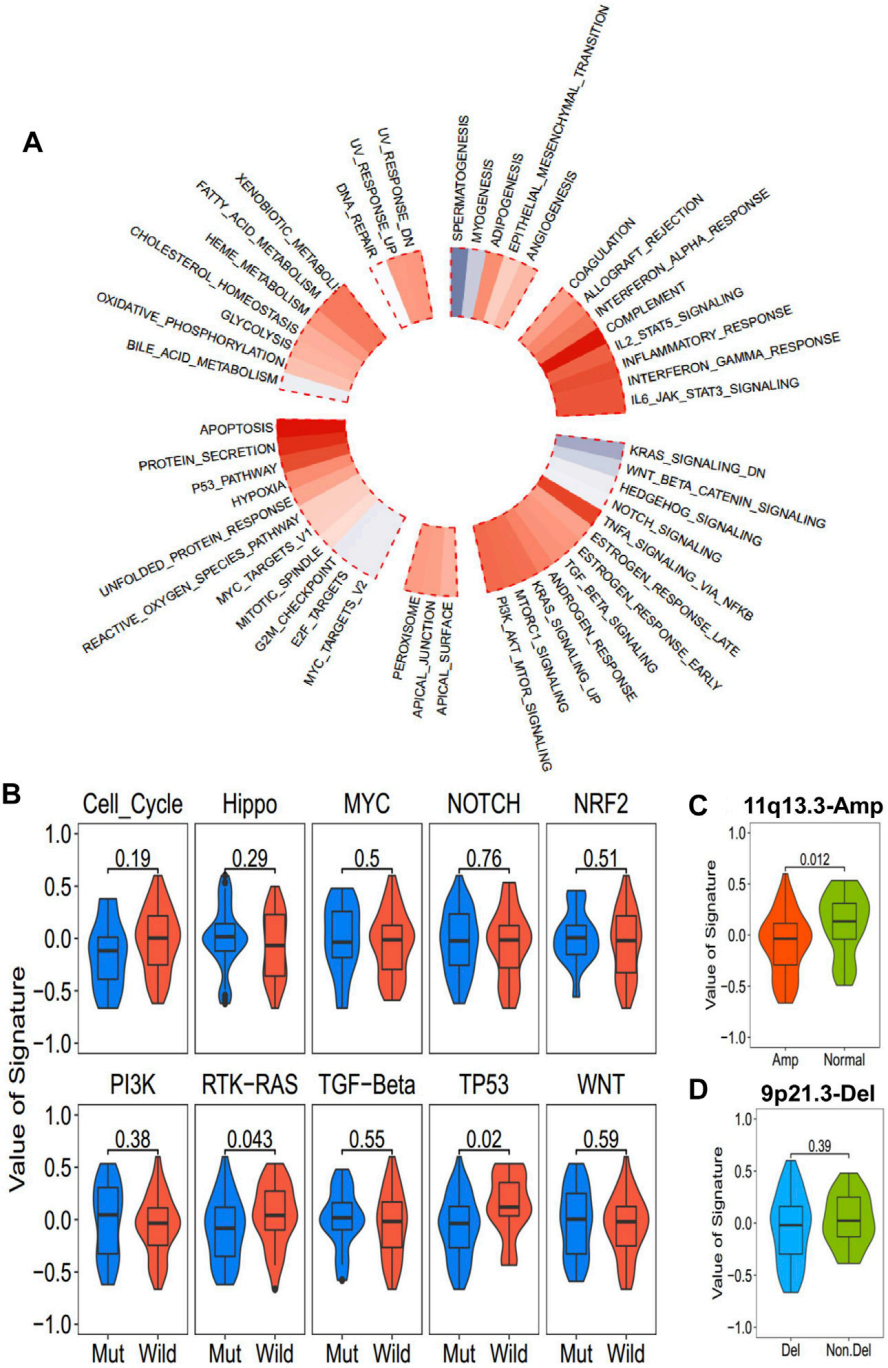
Association between the ICD-score and hallmark pathways. **(A)** Correlation of the ICD score and hallmark pathways (red for positive correlation; gray for negative correlation). **(B)** ICD score of mutated and wild-type groups of hallmark pathways in the TCGA-ESCC cohort. **(C,D)**. ICD score comparison of patients with common copy number alteration in ESCC and normal patients. **(C)** 11q13.3 amplification group vs. normal group. **(D)** 9p21.3 deletion group vs. non-deletion group.

The correlation between ICD-score and hallmark pathways was systematically estimated with the transcriptomic cohort. Apoptosis, protein secretion, TP53, complement, and TNF-alpha signaling *via* NF-kappaB had a strong positive association with the ICD-score, while KRAS signaling, WNT beta catenin signaling, hedgehog signaling, and spermatogenesis were negatively associated with the ICD-score (Figure 6A). Among the 10 oncogenic signaling pathways reported previously (Sanchez-Vega et al., 2018), we found that the TP53 pathway alteration and RTK-RAS alteration were both significantly positively associated with the ICD-score (Figure 6B). Chromosomes 11q13.3 and 9q21.3 were the most two common chromosome alterations in the development of ESCC (Chang et al., 2017; Zhang et al., 2018). In our study, the chromosome 11q13 amplification group exhibited higher ICD-scores than the normal group (*p* = 0.012), while 9q21 deletion showed no statistical difference with the amplicon and normal groups (Figures 6C,D). The result enables us to connect the oncogenic genomic alteration and the ICD-score in ESCC, which would help us to further understand the possible regulated upstream of the ICD subtypes.

### The Predictive Value of ICD-Scores in Immune-Oncology Therapy

**FIGURE 7:**
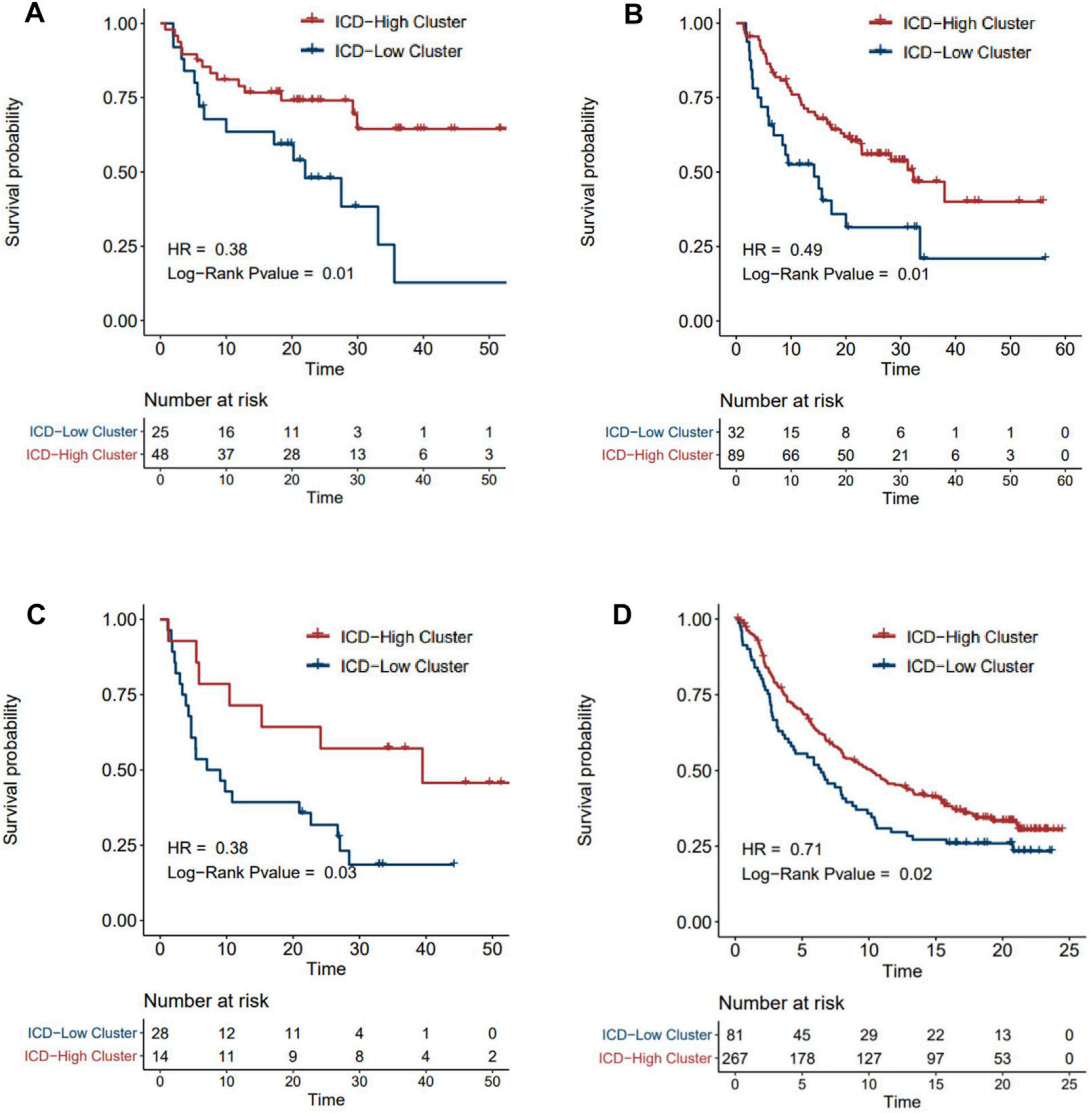
Survival analysis of ICD-score high/low clusters in four immunotherapy cohorts. **(A–D)** Kaplan–Meier analysis of overall survival in Tuba N Gide et al.‘s cohort **(A)**, David Liu et al.‘s cohort **(B)**, Eliezer M Van Allen et al.‘s cohort **(C)**, and Sanjeev Mariathasan et al.‘s cohort **(D)** (red for the ICD-score high cluster; blue for the ICD-score low cluster).

ICI treatment represented by PD-1/CTLA4 inhibitors has undoubtedly caused a major breakthrough in oncology therapy. Considering the strong correlation between ICD subtyping and tumor immune microenvironment, we further investigated whether ICD-score could predict patients’ response to immune checkpoint therapy based on four immunotherapy cohorts. As expected, patients with a higher ICD-score derived significantly longer OS than those with a lower ICD-score (Figures 7A–D), revealing that the ICD-score might be another promising biomarker in immunotherapy.

## Discussion

Accurate molecular typing is an important part of precision medicine in the future. In the past few years, with the development of high-throughput sequencing technology, molecular typing of esophageal cancer based on transcriptome expression data has made great progress. For example, Fengjing Wang et al. obtained two ESCC groups with different clinical survival outcomes from the transcriptome data of 179 ESCC cases and identified the corresponding biomarkers such as FOXA1 and EYA2 for subtype I and LAMC2 and KRT14 for subtype II (Wang et al., 2019b). Meng Liu et al. applied consensus clustering methods on transcriptome data from 125 Xinjiang patients with ESCC to divide the patients into three types and verified the subtypes in two independent cohorts including the TCGA dataset (Liu et al., 2020). It is worth noting that the aforementioned studies were mainly based on the analysis from the whole transcriptome, with limited biological insights, and the discovery dataset was from a single dataset. The main features of our study are that our clustering was inspired by the heterogeneity of ICD in ESCC patients, and the subtypes were obtained by an unsupervised cluster analysis of the patients’ ICD GSVA score from six studies, and these subtypes were further validated and confirmed in an extraproteomic dataset. To our knowledge, the cohort of our study is so far the largest dataset for building ESCC molecular subtypes. Moreover, to facilitate the usage of the ICD scheme in the future, the XGBoost was applied to shrink the gene list representing each ICD group, making a simplified subtyping system. Therefore, our study provides an important immune-associated cell death signal molecular subtyping scheme for ESCC classification in the era of precision medicine.

Regulated cell death (RCD) is an important mechanism for organisms to protect themselves from various diseases and even cancers (Galluzzi et al., 2018). Previous studies have revealed several types of RCDs which were proved to cope with different kinds of adverse conditions. The most common RCDs include intrinsic and extrinsic apoptosis, necroptosis, proptosis, and ferroptosis, which have also been shown to be related to the immunogenic response of tumors (Legrand et al., 2019). In the cancer basic research field, there have been many studies that have explored abnormal different cell death signals in tumors and their crucial roles in tumor immunity. However, in ESCC, the systematic study of interactions between these RCDs and the immune microenvironment has not been carried out yet. In the present study, by performing comprehensive bioinformatics analysis of six published transcriptomic datasets, one proteomic dataset and four ICC treatment cohorts, we characterized all known cell death signals status in ESCCs. With machine learning approaches, we constructed an applicable ICD scoring scheme to classify the ESCC into subtypes, which could be further characterized by distinct genomic features as well as immune profiles. We expected this scheme could be utilized not only in research on the molecular mechanisms governing ESCC but also as a potential prognostic and immunotherapy biomarker for this deadly cancer.

## Data Availability

Publicly available datasets were analyzed in this study. These data can be found at: we have submitted the source data supporting our article to www.jianguoyun.com and shared the download link in Download link of Integrated Data.docx as a Supplementary Material. Information of all published datasets used in the research study is listed in Table 1 in the article. The intermediate files and code are deposited in https://github.com/chenyx47/ESCC_ICD.git/,and the code is also attached with the filename: Code.zip. All the material is available for supporting our article.

## Supplementary Material

Supplementary Table S1 Detail of the genes of the selected cell death pathways and their sources.
