# Supplementary figures and images for: Stratification From Heterogeneity of the Cell-Death Signal Enables Prognosis Prediction and Immune Microenvironment Characterization in Esophageal Squamous Cell Carcinoma
Source: Front Cell Dev Biol. 2022 Apr 12;10:855404. doi: 10.3389/fcell.2022.855404 (PMC9040162; doi:10.3389/fcell.2022.855404)

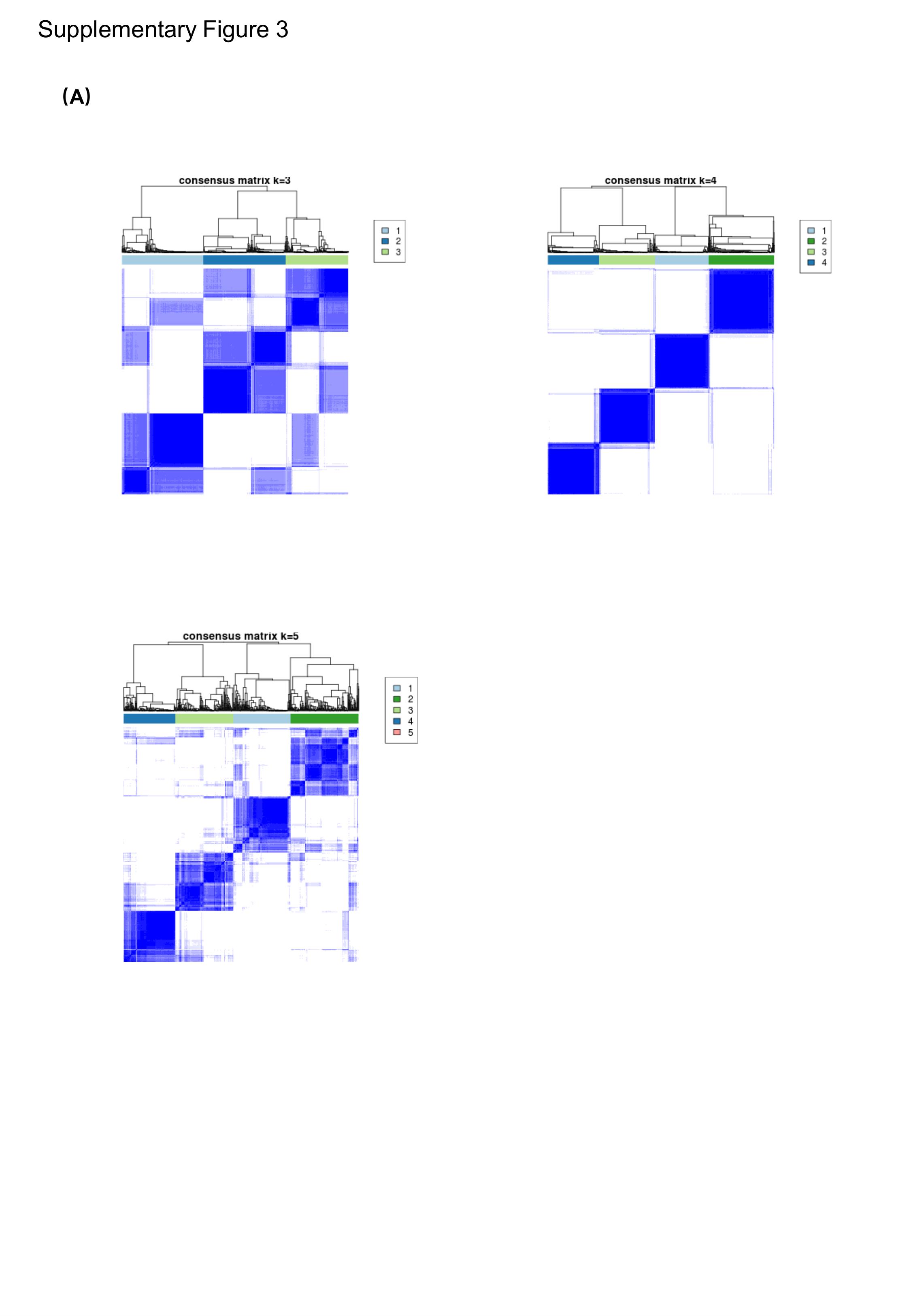

Supplement: Supplementary file 1 [file Image3.JPEG]

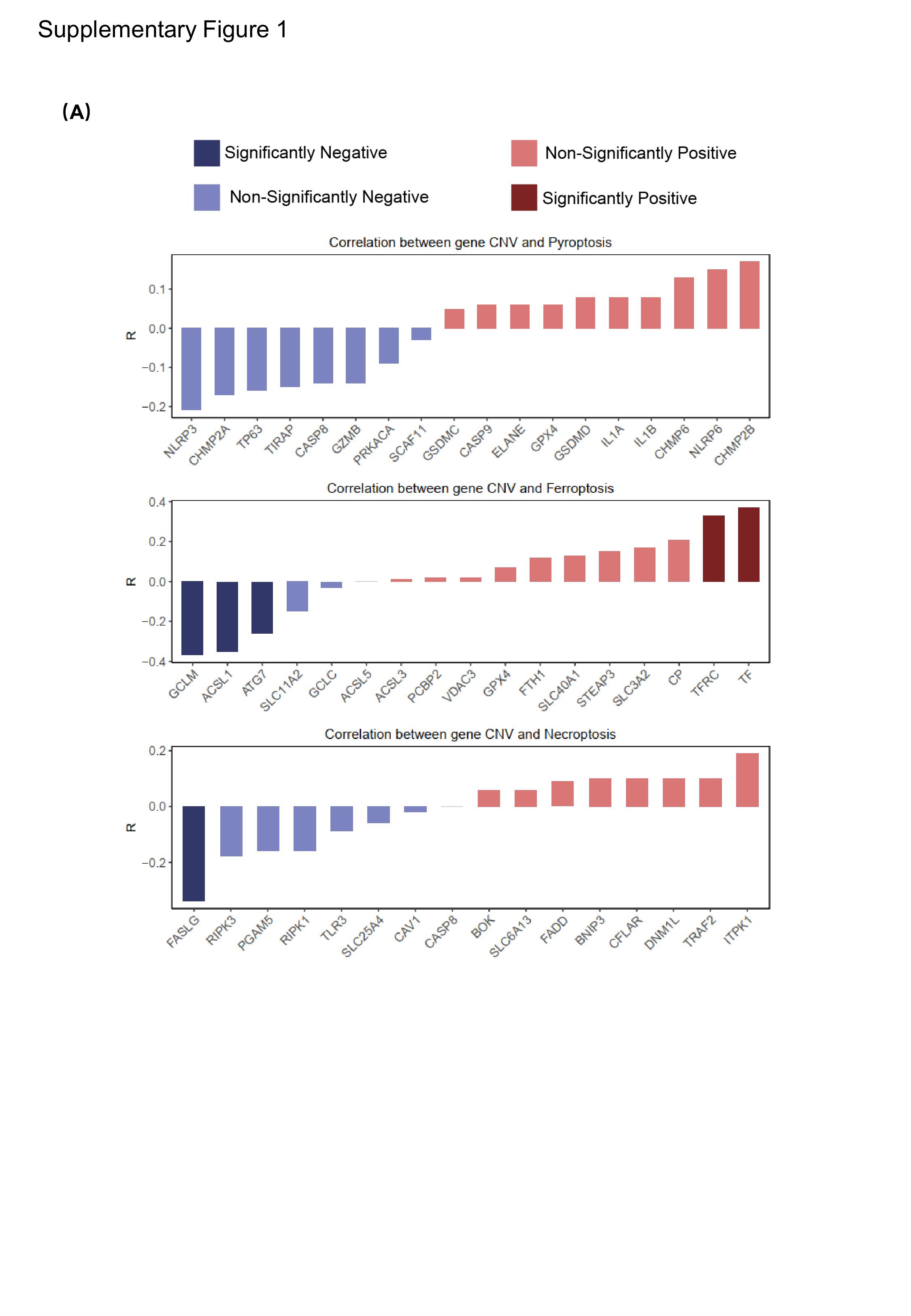

Supplement: Supplementary file 2 [file Image1.JPEG]

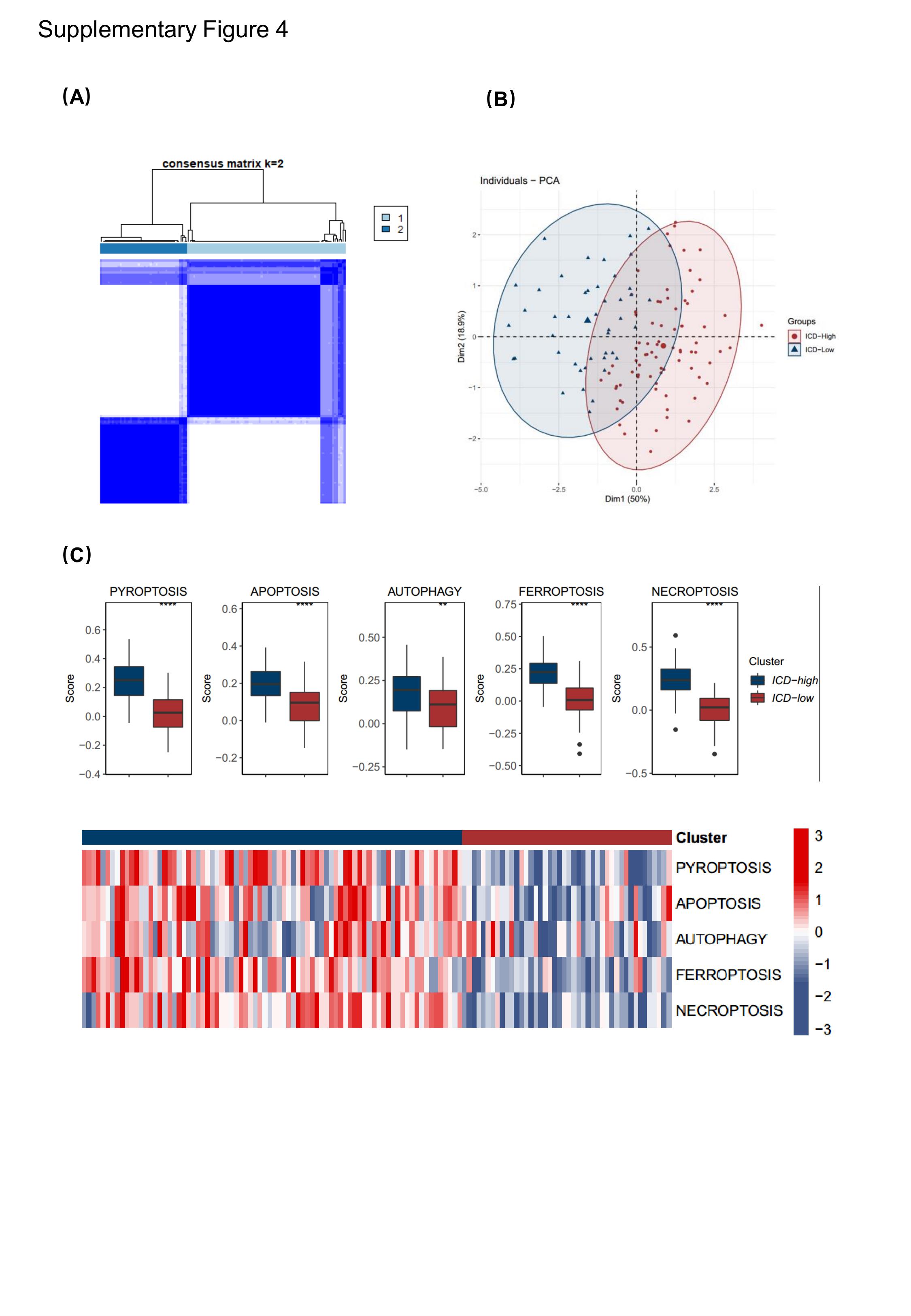

Supplement: Supplementary file 3 [file Image4.JPEG]

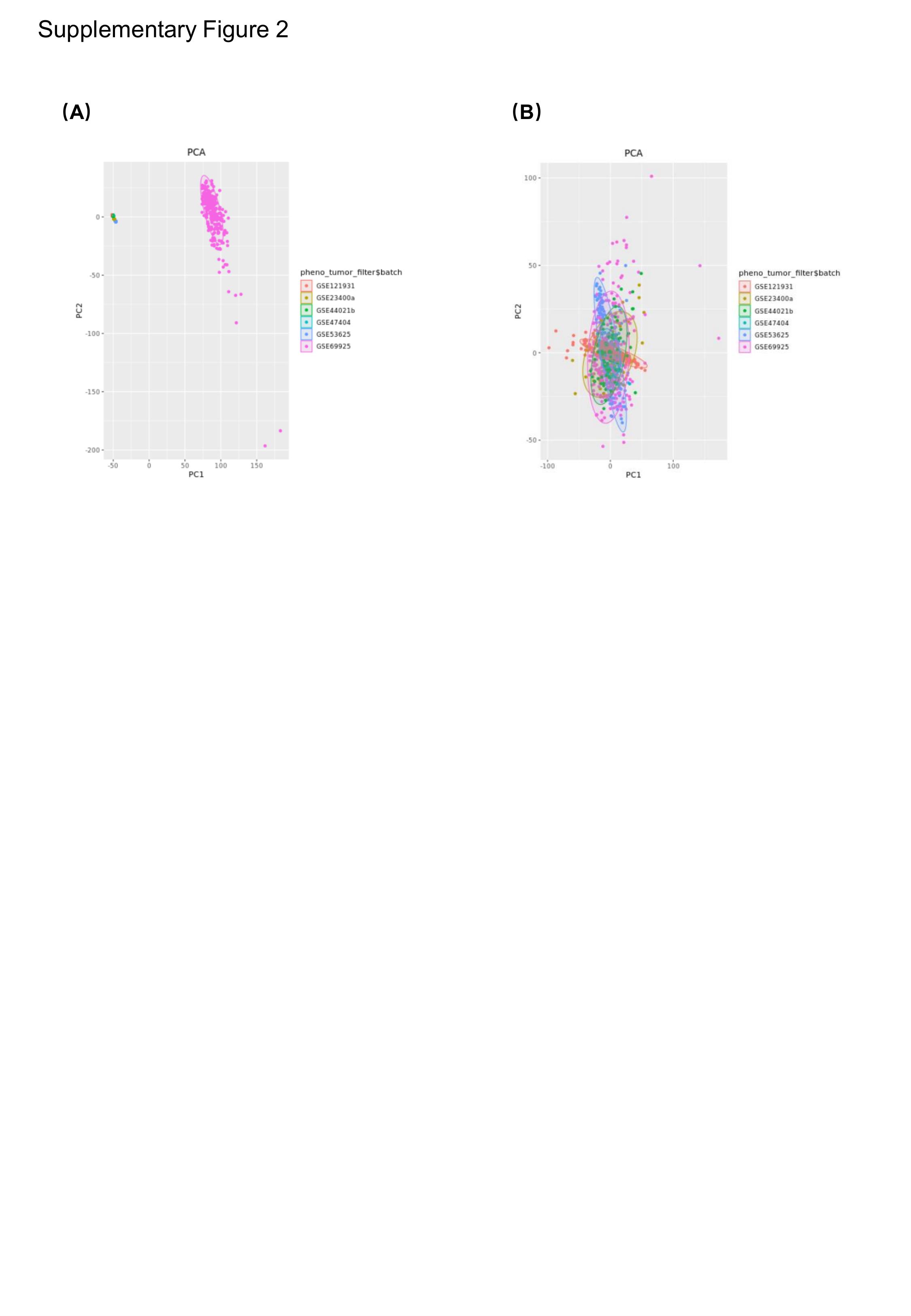

Supplement: Supplementary file 4 [file Image2.JPEG]

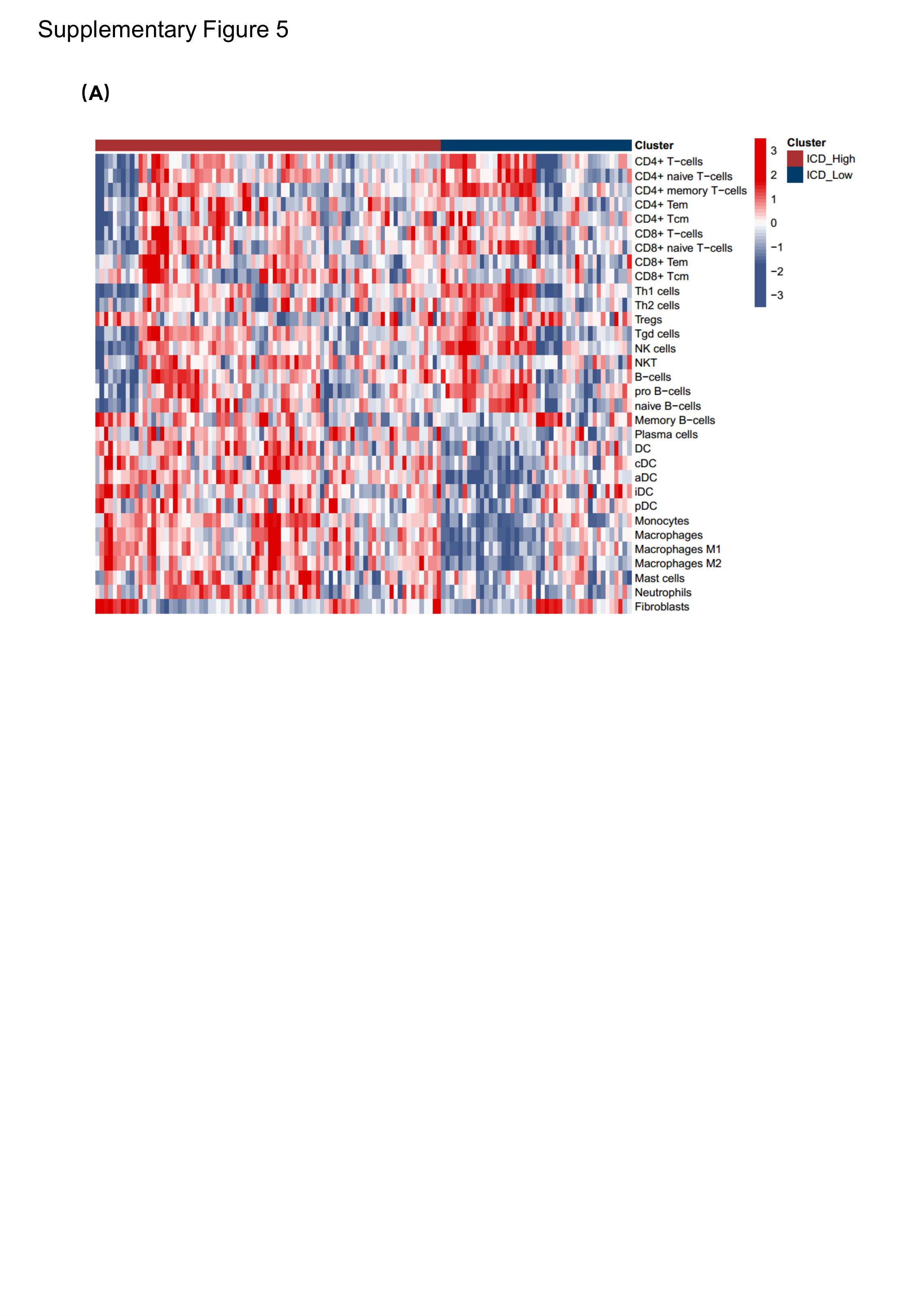

Supplement: Supplementary file 5 [file Image5.JPEG]

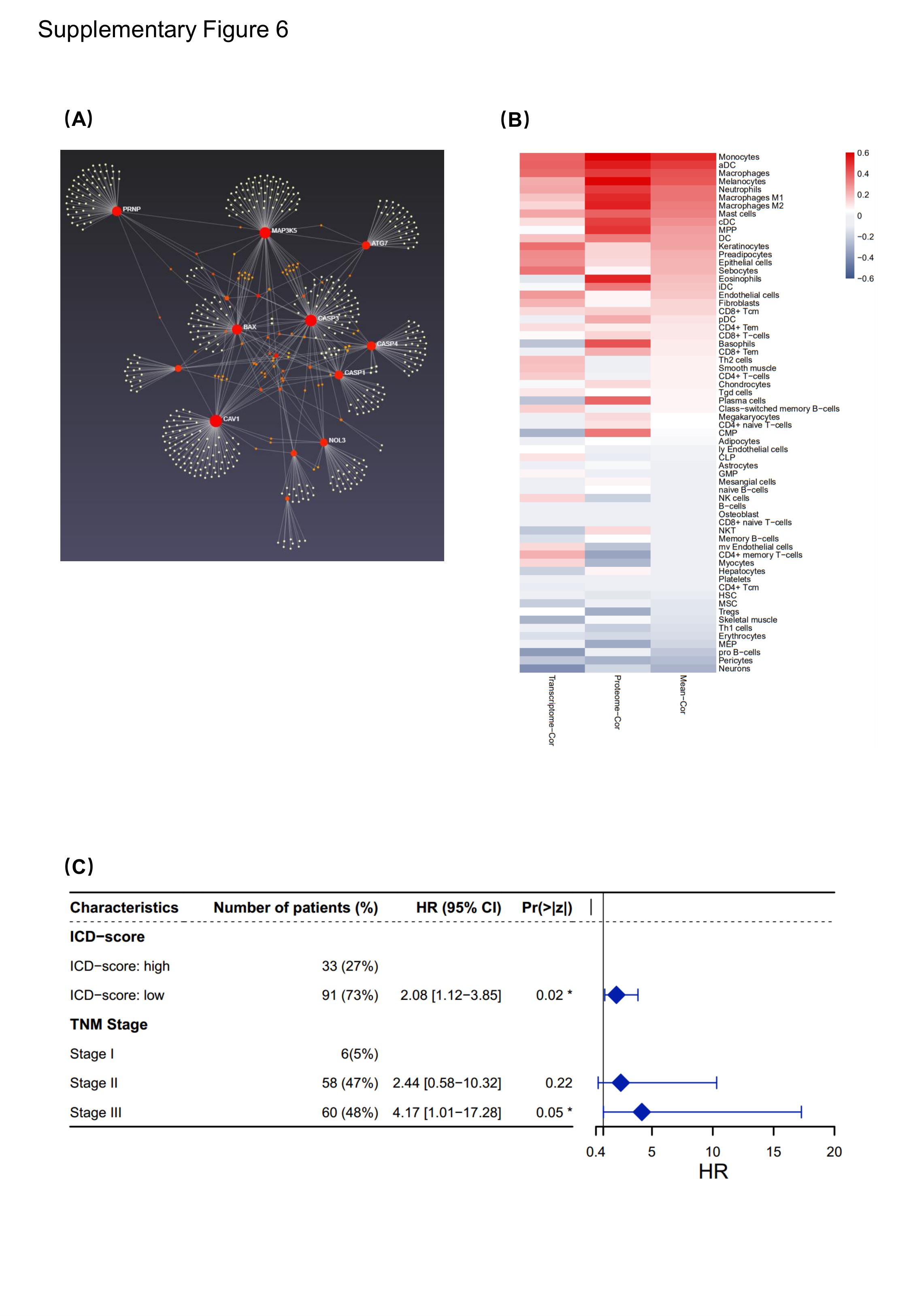

Supplement: Supplementary file 7 [file Image6.JPEG]
